# The Role of Antidiabetic Therapies in Mild Cognitive Impairment and Alzheimer’s Disease: A Systematic Review of Metformin, Pioglitazone, and GLP-1 Receptor Agonists

**DOI:** 10.3390/ijms27093967

**Published:** 2026-04-29

**Authors:** Dina A. Mahoon, Omar Hamad, Alexandra E. Butler

**Affiliations:** 1 School of Medicine, Royal College of Surgeons in Ireland, Medical University of Bahrain, Busaiteen 15503, Bahrain; 22203142@rcsi.com; 2 Research Department, Royal College of Surgeons in Ireland, Medical University of Bahrain, Busaiteen 15503, Bahrain; 3 Royal College of Surgeons in Ireland, Medical University of Bahrain, Busaiteen 15503, Bahrain

**Keywords:** antidiabetic therapy, mild cognitive impairment, Alzheimer’s disease, metformin, pioglitazone, GLP-1 receptor agonists

## Abstract

Alzheimer’s disease (AD) and mild cognitive impairment (MCI) are major causes of cognitive decline. Antidiabetic medications such as metformin, pioglitazone, and GLP-1 receptor agonists have been proposed as potential neuroprotective therapies. We assessed whether these agents slow cognitive decline or disease progression in people with AD or MCI. PubMed, Embase, and Cochrane Central were searched for randomized controlled trials and observational studies of metformin, pioglitazone, or GLP-1 receptor agonists in AD/MCI. Results were synthesized narratively by drug class. Eleven studies met the inclusion criteria. Metformin, particularly in early-stage disease and metabolically vulnerable groups, demonstrated improvements in episodic memory and selective executive outcomes. Observational data in diabetic MCI suggested improved cognition and preservation of hippocampal and cortical structure, with limited amyloid-β and tau changes. Pioglitazone findings varied. Benefits were mainly reported in mild AD with type-2 diabetes, but not in non-diabetic AD/MCI. GLP-1 receptor agonists demonstrated preserved cerebral glucose metabolism and improved blood-to-brain glucose transport but did not improve cognitive function. Current evidence does not support antidiabetic therapies as effective treatments in AD/MCI. Any benefits appear to depend on disease stage and metabolic status, with metformin being the most promising candidate. Larger, longer-duration biomarker-defined trials are needed to determine whether any sustained clinical benefit is observed.

## 1. Introduction

Alzheimer’s disease (AD) is the leading cause of dementia worldwide and a major contributor to cognitive decline in older adults. Clinically, AD is characterized by a gradual onset of episodic memory impairment, followed by progressive deterioration in executive function, visuospatial processing, and other cognitive domains [1,2]. These symptoms reflect the underlying neuropathology of AD, defined by extracellular amyloid-β (Aβ) plaques and intracellular neurofibrillary tangles composed of hyperphosphorylated tau [2].

The global burden of dementia has risen in recent decades, driven by longer life expectancy and ageing populations. Global Burden of Disease data indicate steady increases in incidence, prevalence, and disability-adjusted life years [1,3], with prevalence rising to 33.4% among individuals aged 85 and older [4].

Disease progression often begins years before in AD. Mild cognitive impairment (MCI), particularly the amnestic subtype, is widely regarded as a prodromal stage of AD. Although not all individuals with MCI progress to Alzheimer’s disease, this intermediate state represents a critical window for early identification and potential intervention [5,6].

Despite the scale of the problem, treatment options remain limited [7,8]. This has prompted growing interest in alternative approaches, especially those addressing modifiable biological risk factors.

One such area of interest is metabolic dysfunction. Increasing evidence links AD to impaired insulin and insulin-like growth factor signaling in the brain, sometimes described as brain insulin resistance [9]. Disruption of these pathways affects neuronal energy metabolism and synaptic function and has been associated with amyloid-β accumulation and tau hyperphosphorylation. Metabolic disturbances also promote inflammation and oxidative stress through mechanisms such as advanced glycation product formation, receptor activation, and mitochondrial dysfunction, all of which may accelerate neurodegeneration [9,10].

Together, these observations provide a biological rationale for investigating antidiabetic medications as potential therapeutic strategies in AD and MCI.

### 1.1. Type 2 Diabetes and Alzheimer’s Disease

Type 2 diabetes mellitus (T2DM) is consistently associated with increased risk of developing AD and MCI. Epidemiological studies have reported higher rates of cognitive impairment and dementia in individuals with T2DM [11,12,13]. The risk of developing AD increases by approximately 1.5-fold (RR ≈ 1.56) in diabetic patients compared to non-diabetic populations [14]. This association likely reflects shared metabolic and vascular processes linking T2DM with AD and MCI. Insulin resistance and chronic metabolic stress can impair neuronal function. Diabetes-related microvascular disease also heightens vulnerability to vascular and mixed forms of dementia [9]. In addition, recurrent hypoglycemic episodes in individuals with diabetes may further contribute to neurodegeneration and promote the development of AD [15,16]. Neuroimaging and neuropathological studies demonstrate cerebrovascular and neurodegenerative changes in diabetic populations [17].

Although a definitive causal pathway has not been established, the consistent relationship between metabolic dysfunction and cognitive decline supports targeting metabolic pathways to reduce risk or slow progression in AD and MCI.

### 1.2. Mechanisms of Metformin

Metformin is a first-line therapy for T2DM that lowers blood glucose by suppressing hepatic gluconeogenesis and improving peripheral insulin sensitivity. This is mainly achieved by activating adenosine monophosphate-activated protein kinase (AMPK), a central regulator of cellular energy balance [18]. Activation of AMPK reduces lipogenesis and enhances insulin sensitivity in hepatic and skeletal muscle tissues. Metformin also inhibits mitochondrial complex I, increasing the AMP:ATP ratio and activating AMPK-dependent signalling while exerting AMPK-independent effects on gluconeogenic and redox pathways [18,19]. These mechanisms are relevant to AD and MCI, as impaired insulin signalling, mitochondrial dysfunction, oxidative stress, and chronic low-grade inflammation contribute to neuronal injury and synaptic failure [20].

**Figure 1:**
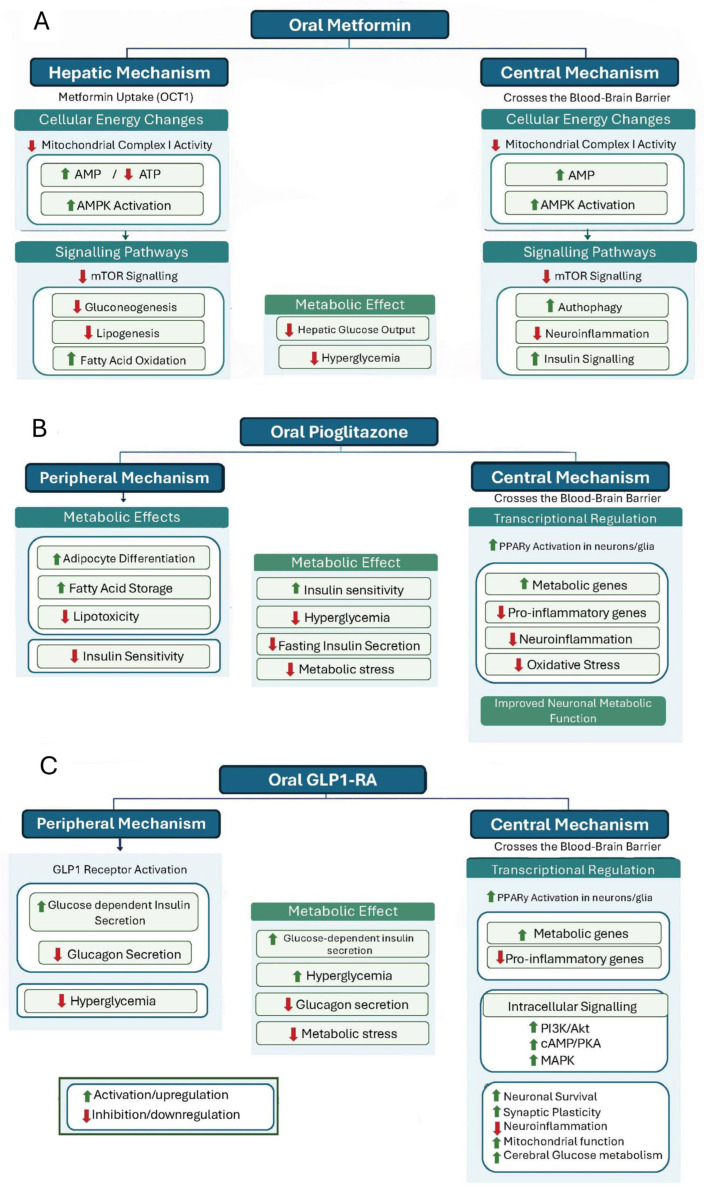
(**A**) Proposed Mechanisms of Metformin in Alzheimer’s Disease and MCI. (**B**) Proposed Mechanisms of Pioglitazone in Alzheimer’s Disease and MCI. (**C**) Proposed Mechanisms of GLP-1RAs in Alzheimer’s Disease and MCI.

In addition to glycemic control, metformin modulates mitochondrial function by partially inhibiting mitochondrial complex I. It also influences mitochondrial respiration and biogenesis through both AMPK-dependent and AMPK-independent pathways [19,21]. Metformin exerts anti-inflammatory effects by activating AMPK and inhibiting the mammalian target of rapamycin (mTOR), which affects mitochondrial activity and autophagy in immune cells [21]. These mechanisms help explain why the impact of metformin may vary across the course of Alzheimer’s disease. Metformin may be more effective in individuals with insulin resistance or T2DM during the early stages of AD, when metabolic dysfunction is a driving factor in neurodegeneration. In contrast, in non-diabetic patients or those with later-stage disease of AD, even if relevant pathways are engaged, clinical benefit may be limited [20]. These actions provide a mechanistic rationale for metformin’s potential relevance in neurodegenerative disease (Figure 1A).

### 1.3. Mechanisms of Pioglitazone

Pioglitazone, a thiazolidinedione insulin sensitizer, acts as an agonist of peroxisome proliferator-activated receptor gamma (PPARγ), a nuclear receptor regulating metabolic and inflammatory gene expression relevant to Alzheimer’s disease pathology. Activation of PPARγ attenuates neuroinflammation by suppressing pro-inflammatory enzymes such as cyclooxygenase-2 and inducible nitric oxide synthase, reducing microglial and astrocytic activation, and lowering cytokines, including interleukin-1β, in experimental models [22,23].

PPARγ activation also regulates genes involved in glucose metabolism, lipid handling and mitochondrial function, linking peripheral insulin sensitization to pathways relevant to neuronal energy balance and inflammatory signalling in AD [23]. This provides a mechanistic basis for the metabolic-status dependency observed in clinical studies, as these effects are more biologically relevant in patients with baseline insulin resistance or T2DM, in whom metabolic dysfunction directly contributes to neuronal stress and cognitive decline [22,23].

In addition to its immunomodulatory effects, pioglitazone influences amyloid processing. Experimental data show that PPARγ activation downregulates β-secretase 1, therefore reducing production and accumulation of amyloid-β, particularly the Aβ1–42 species, in transgenic Alzheimer’s models [24,25]. However, much of this evidence is derived from preclinical systems, and it is still unclear whether therapeutic concentrations in humans are sufficient to reproduce these central effects to the same extent. This may explain why clinical benefits appear more evident in metabolically impaired populations, whereas in non-diabetic patients or later-stage disease, pathway engagement may occur without sufficient impact to modify established neurodegeneration or amyloid burden [22,23,24,25].

The combined anti-inflammatory and amyloid-related effects of pioglitazone provide a biological rationale for its investigation in Alzheimer’s disease and mild cognitive impairment (Figure 1B).

### 1.4. Mechanisms of GLP-1 Receptor Agonists

Glucagon-like peptide-1 receptor agonists (GLP-1 RAs) exert neuroprotective effects by engaging G-protein-coupled receptors (GLP-1R) expressed in key brain regions involved in cognition, including the hippocampus and cortex [26,27]. Upon binding, GLP-1 RAs activate intracellular signalling pathways, especially the cyclic adenosine monophosphate (cAMP)/protein kinase A (PKA) and phosphoinositide 3-kinase (PI3K)/Akt axes, which promote neuronal survival, enhance synaptic plasticity, and support synaptic integrity [26,27].

The metabolic-status dependency observed in clinical studies may reflect the interaction between GLP-1 receptor signalling and impaired insulin pathways in the AD brain. In insulin-resistant states, GLP-1 receptor activation engages downstream signalling pathways linked to neuronal survival and synaptic function, which may remain more responsive in metabolically dysregulated conditions [26,27]. Preclinical evidence supports this, with liraglutide shown to reverse memory impairment, reduce synaptic loss, and decrease amyloid plaque burden in AD models [28].

Regarding central target engagement, GLP-1 RAs such as liraglutide have been shown to cross the blood–brain barrier, although cerebrospinal fluid concentrations in humans are low, reported at approximately 0.02% of plasma levels [27]. Despite this, functional engagement is supported by neuroimaging evidence, with clinical studies demonstrating preservation of cerebral glucose metabolism and improved blood–brain glucose transport in patients with AD [27].

In addition, GLP-1 RAs have been associated with reduced neuroinflammation, oxidative stress, and apoptosis in experimental models of neurodegeneration and cerebral ischaemia [26,27,29]. These effects, alongside evidence of enhanced neuronal survival and synaptic preservation, suggest that GLP-1 RAs act on multiple pathways relevant to neurodegeneration. Their effects are therefore better understood as modulation of neuro-metabolic and neurovascular dysfunction rather than as direct alteration of core AD pathology [26,27,28,29,30].

In addition to the drug classes discussed above, other antidiabetic therapies have also been explored in the context of Alzheimer’s disease. Amylin, a pancreatic peptide co-secreted with insulin, has been implicated in Alzheimer’s disease due to its structural similarity to amyloid-β and its potential role in amyloid aggregation and clearance [31]. Amylin analogs, such as pramlintide, have demonstrated neuroprotective and anti-amyloid effects in preclinical studies, although clinical evidence remains limited. Other approaches, including insulin-based interventions and newer metabolic agents, have also been investigated, but findings remain heterogeneous. Against this broader background, the present review focuses on metformin, pioglitazone, and GLP-1 receptor agonists, which currently have the most developed clinical evidence in MCI and AD.

## 2. Methods

This systematic review was conducted and reported in accordance with the Preferred Reporting Items for Systematic Reviews and Meta-Analyses (PRISMA) guidelines. A prespecified protocol was developed and registered in the International Prospective Register of Systematic Reviews (PROSPERO; CRD420251104390). A comprehensive search was conducted in PubMed (MEDLINE), Embase, and the Cochrane Library.

### 2.1. Eligibility Criteria

Eligibility was defined using the Population, Intervention, Comparator, Outcomes, and Study design (PICOS) framework (Table 1). Searches used controlled vocabulary and keywords for AD/MCI, antidiabetic medications, and cognitive/neurodegenerative outcomes. Searches were limited to human studies published in English from 2000s to the present. Database-specific strategies were applied.

### 2.2. Search String

Search string can be found in Supplementary Table S1.

### 2.3. Study Selection

**Figure 2:**
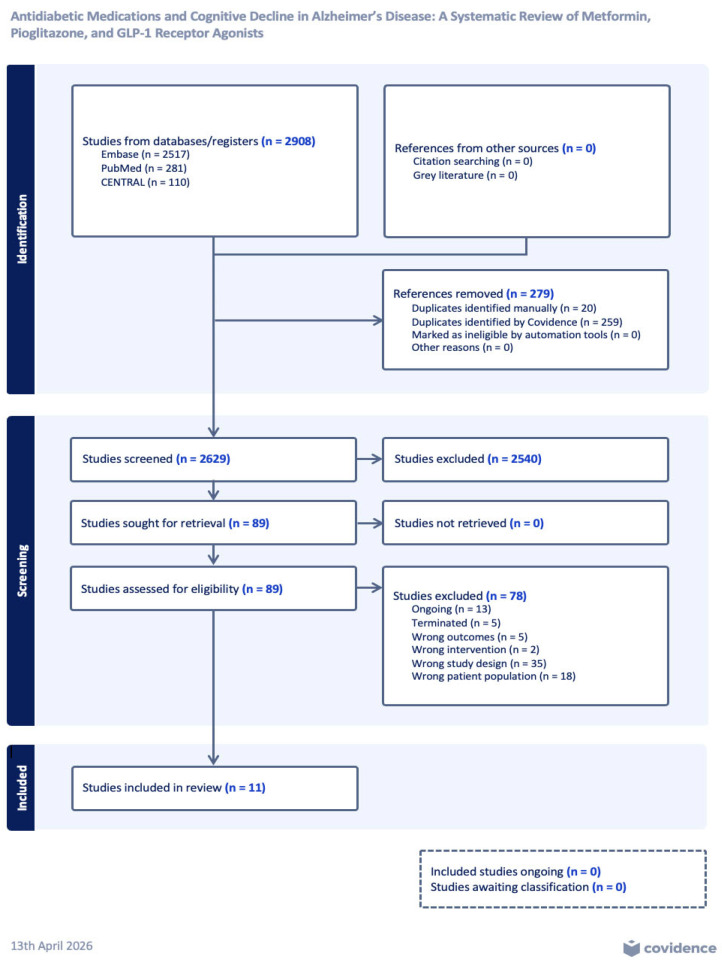
PRISMA Flow Diagram.

All records were imported into Covidence for duplicate removal. Titles and abstracts were independently screened by two reviewers, followed by independent full-text assessment. Disagreements were resolved through discussion and consensus (Figure 2).

### 2.4. Data Extraction

Two reviewers independently extracted data using a standardized form, including study design, setting, sample size, participant demographics, diagnostic criteria, intervention details (agent, dose, duration), comparator, outcome measures and follow-up duration. Discrepancies were resolved by discussion.

### 2.5. Quality Assessment

Two reviewers independently assessed risk of bias. Randomized trials were evaluated using the Cochrane Risk of Bias 2.0 tool, and non-randomized studies were assessed using ROBINS-I (Supplementary Table S2). Disagreements were resolved through discussion.

### 2.6. Data Synthesis

Given substantial heterogeneity in study designs, participant populations, and particularly in reported outcome measures, quantitative pooling was not appropriate. After consultation with a data analyst, it was determined that a meta-analysis was not feasible due to the lack of comparable cognitive and biomarker endpoints across studies. Findings were therefore synthesized narratively and organized by intervention class (metformin, pioglitazone, GLP-1 receptor agonists).

## 3. Results and Discussion

After removing duplicates and screening, eleven studies met inclusion criteria (Figure 2, Table 2). Two publications [32,33] were derived from the same cohort, with the latter representing an extension analysis; these were treated as analytically linked in the synthesis.

The included evidence comprised RCTs (parallel and crossover designs) and one observational cohort study. Study durations ranged from 8 weeks to 18 months. Populations included individuals with amnestic MCI, biomarker-confirmed MCI or early AD, mild to moderate AD, and subgroups with comorbid T2DM or insulin resistance.

Substantial heterogeneity was observed across trials in diagnostic criteria, metabolic status, intervention dosing and duration, cognitive endpoints, imaging modalities, and biomarker assessments. As prespecified, results were synthesized narratively by intervention class.

The characteristics of the included studies are summarized in Table 2.

### 3.1. Metformin

#### 3.1.1. Cognitive Outcomes of Metformin

Across included trials, metformin demonstrated the most consistent cognitive signal among the three drug classes, although effects were domain-specific rather than global.

In overweight adults with amnestic MCI, Luchsinger et al. reported improvement in episodic memory over 12 months, specifically in total recall on the Selective Reminding Test (SRT) [34]. Episodic memory decline often precedes hippocampal dysfunction in prodromal Alzheimer’s disease. Therefore, improvement in this domain may reflect enhanced neuronal function at a stage when cognitive networks are still sufficiently preserved to respond to metabolic intervention. However, no significant between-group difference was observed on the ADAS-Cog. This discrepancy proposes that, if present, metformin’s effect may be subtle and detectable only with sensitive memory-specific instruments rather than composite scales.

A similar domain-restricted pattern was observed in the crossover trial by Koenig et al., where executive performance improved during metformin exposure (Trail Making Test Part B), while other cognitive measures remained largely unchanged [35]. The selective executive improvement, combined with increased orbitofrontal perfusion, suggests short-term functional modulation rather than structural or global cognitive change.

By contrast, in the short-duration biomarker-focused crossover trial by Weinberg et al., no measurable cognitive improvement was detected over eight weeks [37]. This result suggests that either longer exposure is required to observe clinical effects, or that metformin’s cognitive impact may depend on metabolic vulnerability not present in that cohort.

Observational findings from the ADNI cohort further reinforce a stage and context-dependent improvement. In individuals with MCI due to AD and comorbid T2DM, metformin use was associated with better composite cognition and improved memory performance in comparison to diabetic non-users [36]. While causality cannot be inferred, this association suggests that metformin may mitigate metabolically driven cognitive vulnerability in early disease.

#### 3.1.2. Neuroimaging and Biomarker Findings of Metformin

Neuroimaging and biomarker results suggest central biological engagement even when cognitive change is limited.

In the ADNI observational analysis, metformin use was associated with relative preservation of hippocampal volume and cortical thickness in AD-signature regions [36]. These structural findings parallel the memory advantage observed in the same cohort and suggest that metformin may attenuate neurodegenerative processes in metabolically vulnerable individuals.

In the randomized crossover study by Koenig et al., metformin exposure was associated with increased orbitofrontal cerebral blood flow [35]. Although CSF amyloid-β and tau markers remained unchanged over the short treatment period, the perfusion changes suggest functional modulation of frontal networks involved in executive control.

Proteomic analysis by Weinberg et al. demonstrated modulation of inflammatory and apoptosis-related proteins in both plasma and CSF [37]. While classical amyloid and tau biomarkers did not shift, these changes indicate engagement of metabolic and inflammatory pathways relevant to neurodegeneration.

#### 3.1.3. Disease Stage and Metabolic Context of Metformin

Across studies, both disease stage and metabolic status appeared to shape participant response to metformin. The most evident suggestions of cognitive improvement were observed in individuals with amnestic MCI rather than established AD [34,35], and in those with underlying metabolic dysfunction, including insulin resistance or T2DM [34,36]. In Luchsinger et al., stratified analyses suggested stronger effects among participants with lower HbA1c and higher fasting insulin levels [34], further supporting the idea that baseline metabolic vulnerability may influence responsiveness to metformin.

By contrast, in more advanced AD, the cognitive impact of metformin was attenuated and largely limited to selective executive measures [35]. This pattern suggests that metformin may be most effective in earlier stages of disease, when neuronal networks retain enough functional capacity to respond to metabolic modulation. Once neurodegeneration is more established, however, metabolic intervention alone may be insufficient to produce meaningful cognitive improvement.

### 3.2. Pioglitazone

#### 3.2.1. Cognitive Outcomes of Pioglitazone

Across the included studies, pioglitazone produced mixed cognitive outcomes, with results varying by metabolic status and disease stage.

The most favorable results were reported in patients with mild AD and comorbid T2DM [38]. In this subgroup, treatment was associated with improvements in global cognition (MMSE and ADAS-cog Japanese version) and episodic memory (Logical Memory). These changes occurred alongside improvements in insulin sensitivity.

However, this improvement was not appreciated in non-diabetic populations. In mild-to-moderate AD without diabetes, 18 months of pioglitazone failed to produce meaningful differences in global cognition, daily functioning, or neuropsychiatric outcomes compared with placebo [39]. Similarly, in older adults with MCI and insulin resistance, but without established diabetes, pioglitazone improved insulin sensitivity but did not improve cognitive performance [42].

These findings suggest that pioglitazone’s cognitive effects, if present, are contingent upon significant metabolic dysfunction. Improvements in peripheral insulin sensitivity alone may not be sufficient to alter cognitive trajectories in the absence of overt diabetes.

#### 3.2.2. Neuroimaging and Biomarker Findings of Pioglitazone

Neuroimaging and biomarker findings largely mirror the cognitive results. In mild diabetic AD, pioglitazone treatment was associated with improved parietal cerebral blood flow on SPECT imaging [38]. Additionally, the plasma Aβ40/Aβ42 ratio remained stable in the treatment group but worsened in the control group. While plasma biomarkers are indirect measures of central pathology, the stabilization of amyloid-related markers in conjunction with perfusion improvement suggests biological engagement in this subgroup.

In contrast, no consistent neuroimaging or biomarker benefits were observed in non-diabetic AD or MCI populations [39,42]. Structural outcomes and Alzheimer-specific CSF markers did not show significant change.

These results imply that pioglitazone may influence cerebral perfusion in metabolically compromised individuals, but evidence for direct modification of core Alzheimer’s pathology remains limited. own in larger or non-diabetic cohorts, limiting conclusions about disease modification.

#### 3.2.3. Disease Stage and Metabolic Context of Pioglitazone

Across studies, the effect of pioglitazone varied by disease stage and metabolic status. The clearest benefits were observed in patients with mild AD and comorbid T2DM [38]. This pattern suggests that pioglitazone may be more relevant in the presence of metabolic dysfunction, where insulin sensitization could meaningfully influence cognitive outcomes. In contrast, in non-diabetic AD and in MCI characterized by less severe insulin resistance, treatment did not result in measurable cognitive gains [39,42].

Taken together, this pattern implies that pioglitazone may be most effective in populations with significant metabolic impairment, where insulin resistance is more directly implicated in disease expression. In more advanced neurodegeneration, or where metabolic dysfunction is comparatively mild, peripheral insulin sensitization alone may not be sufficient to produce detectable cognitive change.

### 3.3. GLP-1 Receptor Agonists

#### 3.3.1. Cognitive Outcomes of GLP-1 Receptor Agonists

Across clinical trials, GLP-1 receptor agonists have not shown consistent cognitive benefits in Alzheimer’s disease or MCI. In a 26-week double-blind randomized trial of liraglutide in mild to moderate AD, global cognitive outcomes did not differ from placebo, even though liraglutide prevented the expected regional decline in cerebral glucose metabolism [32].

A similar pattern was seen in the 18-month Mullins exenatide trial in early AD, where no significant between-group differences emerged on ADAS-Cog or other neuropsychological measures, and both groups progressed as expected over time [40]. Although reductions in neuronal-derived extracellular vesicle Aβ42 were observed, these biomarker changes did not translate into measurable cognitive benefit. In MCI, a 32-week study of long-acting exenatide likewise failed to demonstrate improvement on global cognitive outcomes [41].

#### 3.3.2. Neuroimaging and Metabolic Findings of GLP-1 Receptor Agonists

Although cognitive outcomes were largely neutral, neuroimaging findings demonstrated clear central effects. In the 26-week liraglutide trial, placebo-treated participants showed the expected decline in cerebral glucose metabolism (CMRglc) in Alzheimer’s-vulnerable regions, including the parietal and temporal cortices, whereas this decline was not observed with liraglutide [32]. Preservation of CMRglc suggests sustained neuronal metabolic function despite the absence of measurable short-term cognitive improvement. In the same cohort, liraglutide increased the maximum blood–brain glucose transport capacity (Tmax), restoring it toward values seen in healthy controls. Baseline Tmax was lower in patients with longer disease duration and was positively associated with cognition. Treatment appeared to reverse this pattern, indicating improved glucose transport at the blood–brain barrier [33].

These metabolic effects were not accompanied by changes in amyloid pathology. PET imaging showed no difference in fibrillary amyloid deposition over six months [40], and in the 18-month exenatide trial, CSF Aβ42, total tau, and phosphorylated tau remained unchanged [40]. Although plasma neuronal extracellular vesicle Aβ42 levels decreased, this isolated biomarker shift was not associated with cognitive or structural improvement.

#### 3.3.3. Disease Stage and Metabolic Context of GLP-1 Receptor Agonists

The disconnect between preserved cerebral glucose metabolism and stable cognition suggests that GLP-1 receptor agonists act primarily through metabolic and neurovascular pathways rather than directly modifying amyloid pathology or producing immediate symptomatic benefit.

In the 26-week liraglutide trial, treatment prevented decline in cerebral glucose metabolism in parietal and temporal regions and restored maximum blood–brain glucose transport capacity (Tmax) toward healthy levels; baseline Tmax was lower with longer disease duration and correlated with cognition [32,33]. Despite these central effects, cognition did not improve, and amyloid PET showed no change in fibrillary amyloid deposition [40].

Similarly, in the 18-month exenatide study, CSF Aβ42, total tau, and phosphorylated tau remained unchanged. Although neuronal-derived extracellular vesicle Aβ42 decreased, this was not associated with structural or cognitive benefit, and clinical progression occurred as expected [40].

Disease stages likely influence response. Once neuronal loss and synaptic dysfunction are established, improving metabolic efficiency may stabilize function without reversing impairment. In MCI, long-acting exenatide showed no overall cognitive improvement, and a treatment-by-sex interaction suggested possible worsening among women, highlighting heterogeneity of response [41].

**Figure 3:**
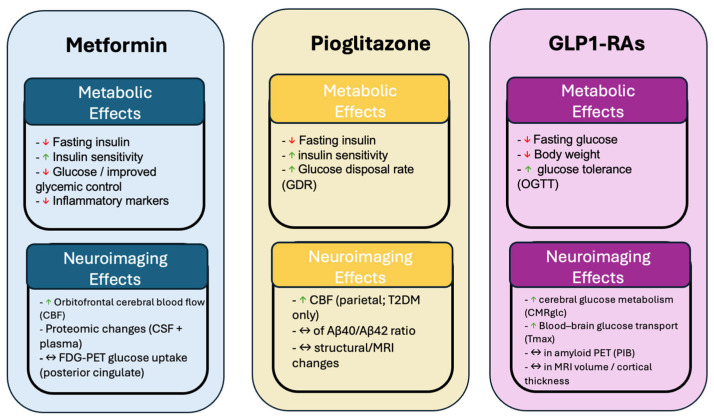
Summary of metabolic and neuroimaging effects of metformin, pioglitazone, and GLP-1 receptor agonists. Upward arrows (↑) indicate an increase, downward arrows (↓) indicate a decrease, and bidirectional arrows indicate no significant change or stabilization.

The cognitive findings across antidiabetic therapies in MCI and Alzheimer’s disease are summarized in Table 3. Metabolic effects and neuroimaging findings can be found in Figure 3.

### 3.4. Progress in Metabolic Therapeutics for Alzheimer’s Disease

Following the influential 2013 review by the Senior group [43], which summarized the early reasons for targeting metabolism in neurodegenerative disease, the treatment field has advanced. It has moved from possible connections to clinical trials confirmed by biological markers. In 2013, the focus was mostly on testing widely used drugs like metformin and thiazolidinediones in large patient groups. Since then, studies from 2013 to 2026 have shown a major shift toward tracking how these drugs affect the brain [32,33,37].

A notable advancement in the field is the emergence of GLP-1 receptor agonists. While this drug class was in its early stages in 2013, recent randomized trials have yielded new findings; specifically, these studies demonstrate its capacity to restore maximal blood-brain glucose transfer and prevent regional declines in cerebral glucose metabolism [32,33,40].

Recent research has advanced the use of high-resolution neuroimaging, proteomics, and neuronal-derived extracellular vesicles. These tools help monitor target engagement within the central nervous system [35,37]. Moreover, this era is characterized by a shift toward metabolic stratification, as researchers now acknowledge that drug efficacy depends greatly on a participant’s baseline insulin resistance and specific disease stage [36,42]. Over the past thirteen years, it has become clear that metabolic pathways in the human brain are now modifiable. However, the gap between restored glucose handling and immediate cognitive improvement remains a significant challenge for the field [41].

### 3.5. Metformin in Alzheimer’s Disease and Mild Cognitive Impairment

Overall, metformin showed the most consistent cognitive effects among the therapies reviewed, although the improvements were modest and largely confined to specific domains. Improvements were seen primarily in episodic memory and executive function [34,35], particularly in early-stage disease and in individuals with insulin resistance or T2DM [22,34]. Rather than producing broad cognitive change, the effects appeared selective, emerging most clearly in recall-based tasks that rely on hippocampal integrity.

Episodic memory decline reflects early hippocampal vulnerability in prodromal Alzheimer’s disease. The finding that total recall improved in amnestic MCI [34] suggests that metformin may exert benefit at a stage when neural networks remain structurally intact but are metabolically strained. In that setting, improving metabolic efficiency may enhance synaptic performance without shifting overall clinical classification. However, the lack of consistent change at global scales, such as the ADAS-Cog, suggests that these effects are subtle. Moreover, repeated cognitive testing can produce practice-related improvements even in placebo groups, potentially masking small treatment effects [34].

Findings from observational cohorts further support this pattern. In the ADNI cohort, metformin use among individuals with MCI and T2DM was associated with better composite memory performance, relative preservation of hippocampal volume, and more favorable CSF profiles [36,37]. While these associations cannot establish causality and may reflect residual confounding, the convergence of structural, biomarker, and cognitive findings strengthens the case that metformin may mitigate metabolically driven vulnerability in early disease.

Imaging and biomarker studies offer additional support. In randomized crossover trials, metformin was linked to increased orbitofrontal cerebral blood flow [35], providing a plausible neural correlate for improved executive performance. Proteomic analyses showed modulation of inflammatory and apoptosis-related pathways in plasma and CSF [37], indicating central biological engagement even when amyloid-β and tau markers remained unchanged over short follow-up [35,37]. Together, these findings suggest that metformin acts primarily through metabolic and inflammatory pathways that support neuronal resilience, rather than directly reducing amyloid deposition.

Importantly, the impact of metformin appears to vary with disease severity. Benefits were more apparent in MCI and early AD [34,35,36]. In more advanced disease, effects were attenuated and largely limited to isolated executive measures [35]. Once neuronal loss becomes substantial, metabolic support alone is unlikely to reverse impairment. This stage-dependent pattern suggests that metformin is unlikely to function as a universal cognitive enhancer but may have relevance as an early adjunctive strategy in metabolically vulnerable populations.

Lastly, it is important to note that the existing studies have clear limitations. The RCTs included are small and brief, limiting power to detect sustained effects [34,35]. Furthermore, heterogeneity in cognitive endpoints complicates comparison across studies, and observational findings are vulnerable to confounding by baseline metabolic control and comorbidity burden [36]. Most trials have also enrolled relatively selected participants, restricting generalizability.

Even with these caveats, a coherent picture emerges. Metformin’s potential appears to be strongest in early-stage disease with demonstrable metabolic dysfunction. The effects are domain-specific and biologically supported by converging imaging and inflammatory data, even in the absence of short-term changes in core Alzheimer’s biomarkers. Larger and longer trials, with prespecified metabolic stratification and sensitive domain-level outcomes, are needed to determine whether these early improvements translate into durable slowing of disease progression.

### 3.6. Pioglitazone in Alzheimer’s Disease and Mild Cognitive Impairment

Pioglitazone’s cognitive effects were less consistent than those observed with metformin and seemed to depend heavily on the presence and severity of metabolic dysfunction. Across the included trials, the most convincing evidence of benefit was limited to individuals with mild Alzheimer’s disease and established T2DM [38]. In this subgroup, improvements in global cognition and episodic memory were accompanied by reductions in fasting insulin and in Homeostatic Model Assessment of Insulin Resistance (HOMA-R), along with increased parietal cerebral perfusion. The fact that cognitive, metabolic, and imaging changes moved in the same direction strengthens the impression that, in the context of clear metabolic dysregulation, insulin sensitization may meaningfully influence brain function. However, this finding should be interpreted cautiously, as Sato et al. was also the only pioglitazone study to report cognitive benefit and used an open-label design, making it more vulnerable to performance and observer bias.

However, this signal did not generalize beyond diabetic cohorts. In non-diabetic patients with mild-to-moderate AD, prolonged treatment did not result in meaningful improvements across cognitive scales, daily functioning, or global status [39]. Similarly, in individuals with MCI and insulin resistance but without diabetes, pioglitazone improved clamp-derived insulin sensitivity, yet these physiological improvements were not accompanied by measurable cognitive progress [39]. This suggests that peripheral insulin sensitization, independently, may not be sufficient to influence cognitive changes unless metabolic dysfunction reaches a threshold that directly contributes to disease expression. Importantly, the two blinded placebo-controlled trials, Geldmacher et al. [39] and Hildreth et al. [42], did not replicate the benefit reported by Sato et al. [38], which further weakens the case for a consistent cognitive effect.

Neuroimaging and biomarker findings reinforce this interpretation. In the mild AD diabetic trial, improved parietal cerebral blood flow and stabilization of plasma Aβ40/Aβ42 ratios were observed with treatment [38], suggesting biological engagement beyond peripheral glycemic correction. In contrast, comparable effects were not observed in non-diabetic cohorts [39,42], in which neither structural imaging nor Alzheimer-specific biomarkers showed consistent changes. Taken together, these findings imply that pioglitazone may influence cerebral perfusion and amyloid-related markers primarily in metabolically compromised individuals.

Disease stage further contextualizes these results. While some improvement was observed in mild AD with T2DM [38], no meaningful benefit was detected in more advanced disease [39]. At later stages, when synaptic loss and neuronal degeneration are more extensive, restoring insulin sensitivity may not reverse established structural damage. Conversely, in early MCI without overt diabetes, metabolic modulation alone may be insufficient to alter cognition over relatively short follow-up periods [42]. This pattern suggests that both the presence of significant metabolic dysfunction and the degree of neurodegeneration shape therapeutic responsiveness.

Several limitations constrain interpretation. All included studies were relatively small, and the most favorable findings arose from an open-label design [38], raising concerns regarding bias. Randomized, placebo-controlled trials in non-diabetic populations did not replicate these benefits [39,42]. Moreover, metabolic heterogeneity across cohorts complicates comparisons. Insulin resistance was more pronounced in diabetic AD cohorts than in MCI populations with milder metabolic disturbance [42], which may explain differential responsiveness.

Based on the current evidence, pioglitazone cannot be viewed as a broadly effective disease-modifying therapy for Alzheimer’s disease or MCI. Rather, its potential appears more limited and context-dependent. The available data point toward a possible role in early-stage Alzheimer’s disease when substantial metabolic dysfunction, particularly insulin resistance or T2DM, is present.

Future trials should reflect this narrower therapeutic window. Rather than enrolling broadly defined AD populations, studies should focus on patients with early-stage disease and clear metabolic impairment. Moreover, longer follow-up periods, combined with serial imaging and biomarker assessments, would help determine whether changes in perfusion or metabolic parameters translate into durable clinical stability. Without this level of clarity and precision in trial design, any real benefit in metabolically defined subgroups is likely to be diluted within heterogeneous samples and ultimately overlooked.

### 3.7. GLP-1 Receptor Agonists in Alzheimer’s Disease and Mild Cognitive Impairment

GLP-1 receptor agonists demonstrated the most robust central metabolic engagement, but the least convincing cognitive translation. Across trials, liraglutide and exenatide preserved cerebral glucose metabolism and improved measures of blood–brain glucose transport [32,33]. Baseline glucose transport correlated with disease duration and cognition [33], suggesting that impaired glucose handling may contribute to progression rather than simply reflect downstream neurodegeneration.

Despite this physiological engagement, cognitive outcomes remained largely unchanged [32,40,41]. In the 26-week liraglutide trial in mild to moderate AD, placebo-treated participants exhibited the expected regional decline in cerebral glucose metabolism, whereas this decline was not observed with treatment; however, global cognitive measures did not differ between groups [32]. Similarly, in the 18-month exenatide trial in early AD, no significant between-group differences were observed on ADAS-Cog or other neuropsychological outcomes, and both groups progressed in line with the natural course of the disease [40]. Although reductions in neuronal-derived extracellular vesicle Aβ42 were observed [40], these biomarker changes did not translate into measurable cognitive or structural benefit.

When evaluated earlier in the disease continuum, results remained neutral. In MCI, long-acting exenatide failed to improve cognition over 32 weeks [41]. ADAS-Cog and other global measures did not shift meaningfully. A treatment-by-sex interaction suggested possible worsening ADAS-Cog trajectories among women receiving exenatide; although the sample size was small and the study was open-label, these findings highlight potential heterogeneity in response and underscore the need for adequately powered, blinded trials [41].

The consistent dissociation between preserved glucose metabolism and unchanged cognition is notable. Restoration of cerebral glucose transport and metabolic activity suggests that neuronal energy utilization remains pharmacologically modifiable even in symptomatic disease. However, these changes may stabilize function rather than reverse established synaptic loss or structural damage. The absence of change in amyloid PET signal and CSF Aβ42, total tau, and phosphorylated tau over six to eighteen months [32,33,40] further suggests that metabolic engagement does not directly modify core pathological aggregates within the timeframes studied.

Disease stage likely influences this pattern. Intervention during symptomatic AD may occur after substantial synaptic and neuronal loss has already taken place, limiting the capacity for functional recovery. Even in MCI, short- to intermediate-duration trials failed to demonstrate cognitive improvement [40,41], raising the possibility that metabolic stabilization requires longer exposure to influence clinical outcomes or that intervention must occur at an even earlier, preclinical stage.

Future trials of GLP-1 receptor agonists should shift earlier in the disease course. Enrolling biomarker-defined preclinical or very early MCI populations, with follow-up extending beyond one to two years, may be necessary to detect meaningful effects. Stratifying participants by the presence and severity of metabolic impairment could also clarify whether those with insulin resistance are more likely to benefit.

### 3.8. Safety and Tolerability Considerations

Safety and tolerability are important when considering the use of antidiabetic therapies in older adults with cognitive impairment. Across the included studies, metformin was generally well tolerated and not associated with serious adverse events. The main issue was gastrointestinal side effects, such as nausea and poor tolerance, which led some participants to reduce the dose or stop treatment altogether [34,35,37]. Pioglitazone also appeared relatively safe, but peripheral edema was reported more frequently, consistent with its known fluid-retention effect [38,39,42]. While this was usually mild, it does raise concerns in older patients, especially those having underlying cardiovascular disease. GLP-1 receptor agonists were similarly well tolerated overall, but gastrointestinal symptoms, including nausea and reduced appetite, were common and sometimes led to discontinuation [32,33,40,41].

From a clinical perspective, these drugs appear reasonably safe, but tolerability may become a limiting factor in this population. Many patients with cognitive impairment are frail, on multiple medications, and more sensitive to side effects. As a result, even mild adverse effects could affect adherence. Consequently, although these therapies are promising, their use in Alzheimer’s disease will demand rigorous patient selection, gradual dose titration, and vigilant long-term monitoring to optimize outcomes and minimize risk.

## 4. Conclusions

This systematic review evaluated three major antidiabetic drug classes in mild cognitive impairment and Alzheimer’s disease. Although metabolic dysfunction is closely associated with neurodegeneration, targeting metabolic pathways alone has not yet translated into meaningful cognitive improvement in established disease.

Among the agents reviewed, metformin demonstrated the most consistent signal of domain-specific benefit, particularly in early-stage disease and in individuals with insulin resistance or T2DM. Effects were most evident in episodic memory and, to a lesser extent, executive function rather than global cognitive scales. Pioglitazone showed selective benefits in metabolically vulnerable subgroups but largely neutral results in non-diabetic populations and more advanced disease. GLP-1 receptor agonists engaged central metabolic pathways and preserved cerebral glucose transport or metabolism; however, cognitive outcomes remained largely unchanged.

Collectively, these findings suggest that antidiabetic therapies are unlikely to function as direct disease-modifying agents in Alzheimer’s disease. Their potential role may instead lie as targeted adjuncts in early-stage or metabolically at-risk populations, where disease stage and metabolic context influence response.

The current evidence base is preliminary. Trials are small, heterogeneous, and often underpowered for cognitive endpoints, and follow-up durations may be insufficient to capture delayed effects of metabolic modulation. Larger, longer-duration studies in earlier, biomarker-defined populations with metabolic stratification and sensitive domain-level cognitive measures are needed. Until such data are available, antidiabetic therapies cannot be recommended as routine cognitive treatments in MCI or Alzheimer’s disease, though metabolic vulnerability remains an important avenue for continued investigation.

## Figures and Tables

**Table 1:** Inclusion and Exclusion Criteria.

PICO Component	Inclusion Criteria	Exclusion Criteria
Population (P)	1—Adults (≥18 years) diagnosed with Alzheimer’s disease (AD) or mild cognitive impairment (MCI) based on recognized clinical criteria (e.g., NINCDS-ADRDA, DSM, Petersen’s criteria) 2—May have comorbid type 2 diabetes mellitus, but AD or MCI must be the primary diagnosis 3—Participants must be receiving at least one of the interventions of interest and report relevant cognitive or neurodegenerative outcomes	1—Animal or in vitro studies 2—Participants without AD or MCI as the primary diagnosis 3—Studies including only other types of dementia (e.g., vascular dementia, Lewy body dementia, frontotemporal dementia) 4—Paediatric populations (<18 years)
Intervention (I)	1—Metformin 2—Pioglitazone 3—Glucagon-like Peptide-1 (GLP-1) receptor agonists	1—Other antidiabetic agents not listed in inclusion 2—Combination therapy where the effect of the listed drug cannot be separated from other medications
Comparator (C)	1—Usual care 2—Placebo 3—No intervention	Comparisons only between two active drugs of interest (e.g., metformin vs. pioglitazone) without a placebo or usual care arm
Outcomes (O)	1—Quantitative measures of cognitive function (e.g., MMSE, ADAS-Cog, MoCA) 2—AD progression or conversion from MCI to AD 3—Neuroimaging or neurodegeneration biomarkers (e.g., MRI volumetrics, PET amyloid/tau, CSF biomarkers)	1—Outcomes unrelated to cognition or neurodegeneration (e.g., only metabolic/glycemic outcomes without cognitive data) 2—Studies reporting only quality-of-life or functional measures without cognitive endpoints
Study Characteristics (S)	1—Randomized controlled trials (RCTs) 2—Prospective or retrospective cohort studies 3—Case–control studies	1—Systematic reviews, meta-analyses, narrative reviews 2—Case reports, case series 3—Conference abstracts without full data 4—Editorials, commentaries, letters to the editor

**Table 2:** Study Characteristics of Included Studies.

Study	Design	Population	Sample Size	Drug	Comparator	Dose, Frequency	Follow Up Duration	Primary Outcomes	Secondary Outcomes
Luchsinger et al. [34]	RCT, Parallel Group	Overweight/obese adults (BMI ≥ 25) with amnestic MCI	80	Metformin	Placebo	Titrated from 500 mg once daily to 1000 mg twice daily over a 4-week period	12 months	Buschke Selective Reminding Test (SRT)	ADAS-Cog; other cognitive domains; tolerability
Koenig et al. [35]	RCT, Crossover Group	Non-diabetic adults with MCI or early dementia due to AD and ≥1 positive AD biomarker	20	Metformin	Placebo	500 mg twice daily (1000 mg/day total)	8 weeks	Executive function (Trail Making Test Part B)	Cerebral blood flow; CSF Aβ/tau; metabolic parameters
Pomilio et al. [36]	Observational Study	Individuals with MCI due to Alzheimer’s disease and comorbid type 2 diabetes	861	Metformin	Standard anti-diabetic therapy	NR	NA	Cognitive composite scores (memory-focused)	Hippocampal volume; cortical thickness; CSF biomarkers
Weinberg et al. [37]	RCT, Crossover Group	Adults with biomarker-confirmed MCI or early AD dementia; no diabetes/pre-diabetes	20	Metformin	Placebo	500 mg daily, titrated weekly to 2000 mg/day (BID)	16 weeks	Changes in plasma and CSF proteomic markers	Plasma/CSF proteomics; inflammatory markers
Sato et al. [38]	Prospective, randomized, open-controlled trial	Adults with mild Alzheimer’s disease (AD) accompanied by type II diabetes mellitus	42	Pioglitazone	Standard anti-diabetic therapy	Pioglitazone 15–30 mg daily	6 months	Global cognition (MMSE, ADAS-cog Japanese version)	rCBF (SPECT); plasma Aβ ratio; HOMA-IR
Geldmacher et al. [39]	RCT, Parallel Group	Nondiabetic adults with probable Alzheimer’s disease (MMSE 12–26, CDR 1–2)	29	Pioglitazone	Placebo	15–45 mg/day, titrated (once daily)	18 months	ADAS-Cog Score	CDR-SB; ADL; neuropsychiatric symptoms; safety
Hildreth et al. [32]	RCT, Parallel Group	Older adults with MCI and insulin resistance	78	Pioglitazone	Placebo	30 mg/day for 1 month, then 45 mg/day (as tolerated)	6 months	Insulin sensitivity (glucose disposal rate via clamp)	Cognitive composites; fasting insulin; metabolic measures
Gejl et al. [32,33]	RCT, Parallel Group	Non-diabetic adults with clinically diagnosed Alzheimer’s disease	38	Liraglutide	Placebo	0.6–1.8 mg/day (titrated weekly; once daily)	26 Weeks	Amyloid deposition (PIB PET)	FDG-PET CMRglc; cognitive testing; blood pressure
Mullins et al. [40]	RCT, Parallel Group	Non-diabetic patients with MCI or mild Alzheimer’s disease with biomarker-confirmed AD	27	Exenatide	Placebo	5 µg BID for 1 week, then 10 µg BID (SC)	18 months	Tmax (blood–brain glucose transport capacity)	CSF Aβ/tau; MRI volumetrics; EV biomarkers
Dei Cas et al. [41]	RCT, Parallel Group	Non-diabetic patients with mild cognitive impairment	32	Exenatide (long-acting)	No Treatment	2 mg SC weekly	32 Weeks	ADAS-Cog Score	IADL; semantic fluency; sex interaction

**Table 3:** Cognitive Outcomes Summary Across Antidiabetic Therapies in MCI and Alzheimer’s Disease.

Study	Drug	Population	Duration	Global Cognition	Episodic Memory	Executive Function	Attention/Other Domains	Overall Cognitive Interpretation
Luchsinger et al. [34]	Metformin	Overweight adults with amnestic MCI	12 months	No significant treatment effect	Significant improvement in total recall on Selective Reminding Test	No significant between-group difference	No significant differences across other domains	Domain-specific benefit in episodic memory
Koenig et al. [35]	Metformin	Non-diabetic adults with MCI or mild AD	8 weeks (crossover)	No significant treatment effect	Non-significant directional improvement	Significant improvement in executive function (Trail Making Test B)	Non-significant directional changes in attention	Short-term executive-domain signal only
Pomilio et al. [36]	Metformin	MCI with comorbid T2DM	Observational	Better composite cognition in metformin users	Better memory composite in metformin user	Not clearly separated by domain	Not reported	Favorable association in diabetic MCI (observational; non-causal)
Weinberg et al. [37]	Metformin	Biomarker-confirmed MCI or early AD	8 weeks (crossover)	No significant treatment effect	No significant treatment effect	No significant treatment effect	No significant treatment effec	Primarily biomarker-focused study; no cognitive benefit detected
Sato et al. [38]	Pioglitazone	Mild AD with T2DM	6 months	Improvement in MMSE and ADAS-Jcog within treatment group	Improvement in Logical Memory within treatment group	No significant treatment effect	No significant change in verbal fluency	Cognitive improvement observed in diabetic mild AD (open-label design)
Geldmacher et al. [39]	Pioglitazone	Nondiabetic AD	18 months	No significant treatment effect on ADAS-Cog t	Not separately reported	Not separately reported	Not separately reported	No evidence of cognitive efficacy
Hildreth et al. [32]	Pioglitazone	MCI with insulin resistance	6 months	No significant between-group difference	No significant between-group difference	No significant between-group difference	No significant between-group difference	No cognitive benefit despite improved insulin sensitivity
Gejl et al. [32,33]	Liraglutide (GLP-1RA)	Non-diabetic AD	26 weeks	No significant treatment effect	No significant treatment effect	No significant treatment effect	No significant treatment effect	Preservation of cerebral glucose metabolism without cognitive improvement
Mullins et al. [40]	Exenatide (GLP-1RA)	Biomarker-confirmed early AD	18 months	No significant treatment effect	No significant treatment effect	No consistent effect	No significant treatment effect	No cognitive efficacy signal
Dei Cas et al. [41]	Exenatide (GLP-1RA)	MCI	32 weeks	No significant treatment effect	No significant treatment effect	No significant treatment effect	Possible treatment-by-sex interaction	No overall cognitive benefit

## Data Availability

The original contributions presented in this study are included in the article. Further inquiries can be directed to the corresponding author.

## Supplementary Materials

The following supporting information can be downloaded at: https://www.mdpi.com/article/10.3390/ijms27093967/s1.
