# Supplementary material for: The Role of Antidiabetic Therapies in Mild Cognitive Impairment and Alzheimer’s Disease: A Systematic Review of Metformin, Pioglitazone, and GLP-1 Receptor Agonists
Source: Int J Mol Sci. 2026 Apr 29;27(9):3967. doi: 10.3390/ijms27093967 (PMC13163320; doi:10.3390/ijms27093967)
Supplement: Supplementary file 1 [file ijms-27-03967-s001.zip › ijms-4221360-supplementary.pdf]

Supplementary Table S1. MeSH and Non-MeSH Search String

Search String	
**MeSH**	**Non-MeSH**
(("Alzheimer Disease"[Mesh] OR "Mild Cognitive Impairment"[Mesh]) AND ("Metformin"[Mesh] OR "Pioglitazone"[Mesh] OR "Glucagon-Like Peptide 1"[Mesh]) AND ("Cognition Disorders"[Mesh] OR "Disease Progression"[Mesh] OR "Neuroimaging"[Mesh])) AND ("Humans"[Mesh]) AND (English[lang]).	(("Alzheimer's disease"[Title/Abstract] OR AD[Title/Abstract] OR "mild cognitive impairment"[Title/Abstract] OR MCI[Title/Abstract] OR "mild cognitive decline"[Title/Abstract]) AND (metformin[Title/Abstract] OR pioglitazone[Title/Abstract] OR "GLP-1 receptor agonist"[Title/Abstract] OR liraglutide[Title/Abstract] OR exenatide[Title/Abstract] OR dulaglutide[Title/Abstract] OR semaglutide[Title/Abstract]) AND ("cognitive function"[Title/Abstract] OR "cognitive decline"[Title/Abstract] OR memory[Title/Abstract] OR "executive function"[Title/Abstract] OR progression[Title/Abstract] OR "conversion to dementia"[Title/Abstract] OR MRI[Title/Abstract] OR PET[Title/Abstract] OR biomarkers[Title/Abstract] OR neurodegeneration[Title/Abstract])) AND (English[lang]).

Supplementary Table S2. Risk of Bias Assessment of Included Studies

A. Randomized Controlled Trials (RoB 2)								
**Study (Author, Year)**	**Sequence Generation**	**Allocation Concealment**	**Blinding of Participant/ Personnel**	**Blinding of Outcomes**	**Incomplete Outcome Data**	**Selective reporting**	**Other Sources of Bias**	**Overall**
Luchsinger et al., 2016	Low	Low	Low	Low	Low	Low	Low	Low
Koenig et al., 2017	Low	Low	Low	Low	High	Low	Low	High
Weinberg et al., 2024	Low	Unclear	Low	Low	Low	Unclear	Unclear	Some concerns
Sato et al., 2011	Unclear	Unclear	High	High	Low	Unclear	Unclear	High
Geldmacher et al., 2011	Unclear	Low	Unclear	Low	Low	Low	Low	Some concerns
Hildreth et al., 2015	Low	Low	Low	Low	Low	Low	Low	Low
Gejl et al., 2016; 2017	Low	Low	Low	Low	Unclear	Low	Low	Some concerns
Mullins et al., 2019	Unclear	Unclear	Low	Low	High	High	High	High
Dei Cas et al., 2024	Low	Unclear	High	Low	Unclear	Low	Unclear	High
**B. Observational Studies (ROBINS-I)**								
**Study**	**Confounding**	**Selection**	**Classification**	**Deviations**	**Missing Data**	**Measurement**	**Reporting**	**Overall**
Pomilio et al., 2022	Serious	Moderate	Low	Low	Moderate	Low	Moderate	Serious
